# The National Pension Fund

**Published:** 1887-09-03

**Authors:** 


### The National Pension Fund.

It will save a good deal of trouble if nurses and other hospital officials who propose joining the National Pension Fund will send their names and other particulars to *the Secretary of The Hospitals Association*, Norfolk House, Norfolk Street, W.C., and not to the Editor of The Hospital. Names continue to arrive in considerable numbers, but if those interested desire the Fund to be started before the end of the Jubilee year they must influence and interest all their friends immediately.

Readers will please note that in sending in their names, nurses and other hospital officials are not committing themselves to join any particular Fund, but simply stating their willingness to join "a Pension Fund if the rules and regulations are such as they approve."

### From a "Hospital" Reader.

I am much interested in the idea of forming a "National Pension Fund for Trained Nurses," which must be my apology for thus writing to you. "The Women's Jubilee Offering" is to be given in some way for the benefit of nurses. Will not some of your readers advocate that part of the money should be devoted to the Pension Fund? then no doubt the Fund might be started before the end of the Jubilee year, a consummation which many nurses earnestly desire.

### From a Hospital Nurse.

I have noticed with interest various notices appearing in The Hospital paper with regard to pensions for nurses. Like many of your correspondents I sometimes look forward and wonder, if disabled, what I should do. I should like to join some Society, such as I imagine you think of originating. Could you furnish me with a list of rules as to how, etc., the subscriptions will be paid; also in what cases and how much benefit is to be derived from the Society? I am deeply interested in this, and shall feel obliged by a speedy reply.

Miss L. Watson Tulloch writes:—"To-day, while reading The Hospital of August 27th, 1887, I see that Mrs. Bedingfeld, Lady Superintendent of the Belgravia Institute, says, 'Would it not be a good thing to bring the proposed National Pension Fund for Nurses before the Queen?' I thought it might interest you and the readers of The Hospital to know that on the 19th inst. I read in the *Standard* that the Queen intended to devote the surplus of the Women's Jubilee Offering for the benefit of nurses or nursing establishments. Whereupon I wrote to the Queen, and suggested to her Majesty that part of the money might go to establishing the National Pension Fund for disabled nurses, or a home of rest, or a home hospital for sick nurses. And I am much pleased to state that on the 22nd inst. her Majesty caused a reply to be sent to me, stating that the question to which my letter alluded has been referred for the consideration of a special committee. So we will yet hope that this year of Jubilee may see the National Pension Fund started."

